# Influence of α‐Linolenic Acid on the Intestinal Barrier Integrity and Intestinal Antioxidant Status in Broilers

**DOI:** 10.1002/fsn3.70271

**Published:** 2025-05-28

**Authors:** Ao Kang, Jialei Ni, Xinyu Cheng, Shuyu Wu, Yun Liu, Weiming Ma, Dong Wang

**Affiliations:** ^1^ College of Veterinary Medicine, Shandong Agricultural University Tai'an Shandong People's Republic of China; ^2^ College of Veterinary Medicine Northeast Agricultural University Harbin Heolongjiang People's Republic of China

**Keywords:** α‐linolenic acid, broiler, growth performance, intestinal antioxidant status, intestinal barrier function

## Abstract

This study aimed to investigate the beneficial effects of α‐linolenic acid (ALA) on intestinal barrier function and antioxidant status in broilers, along with the associated molecular mechanisms. 320 one‐day‐old Arbor Acres broilers were randomly divided into four groups, each with eight replicates, and fed diets with 0 (control), 200, 400, and 600 mg of ALA/kg for 42 days. ALA supplementation did not significantly affect the broilers' overall growth performance. Supplementing diets with 400 and 600 mg/kg of ALA significantly enhanced (*p* < 0.05) jejunal and ileal villus height, the jejunal villus height to crypt depth ratio, and ileal mRNA expression and protein levels of Zonula occludens‐1 (ZO‐1) and occludin in broilers on Day 42. Broilers fed diets containing 600 mg/kg of ALA exhibited significantly increased (*p* < 0.05) serum catalase (CAT) activity, total antioxidant capacity (T‐AOC), and jejunal and ileal activities of CAT and total superoxide dismutase (T‐SOD), alongside reduced malondialdehyde (MDA) concentrations in serum, jejunum, and ileum on Days 21 and 42, compared to the control group. Supplementing 600 mg/kg of ALA significantly increased (*p* < 0.05) the mRNA expressions of CAT, SOD1, NRF2, and HO‐1, along with the protein levels of cytoplasmic and nuclear NRF2 and HO‐1 in the jejunum and ileum on Days 21 and 42. These findings demonstrate the protective effects of ALA in improving intestinal health in broilers. The underlying mechanisms may involve enhancing intestinal barrier integrity by increasing tight junction protein abundance and boosting intestinal antioxidant capacity by elevating antioxidant enzyme activity and activating the NRF2 pathway. In conclusion, our results showed that 600 mg/kg of ALA was identified as the optimal concentration for improving intestinal barrier function and antioxidant status in broilers, highlighting its potential for protecting intestinal health through ALA‐based interventions.

## Graphical abstract

A summary of the findings and proposed mechanism of dietary α‐linolenic acid on the intestinal barrier integrity and intestinal antioxidant status in broilers. In this study, we discovered that the administration of ALA effectively enhances intestinal barrier function and improves intestinal antioxidant status in broilers. These effects were attributed to the upregulation of intestinal antioxidant enzyme activity and activation of the NRF2 pathway. The present study is the first to demonstrate the effect of ALA supplementation on enhancing intestinal antioxidant capacity and promoting intestinal health in broilers.

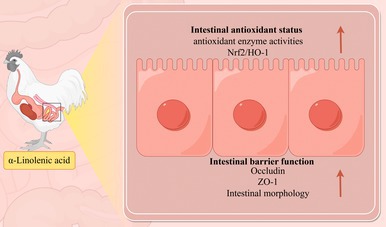

## 1 Introduction

An intact intestinal mucosa is essential not only for nutrient digestion and absorption but also as an innate defense barrier against pathogenic infections (Chairakaki and Greece 2009; Xie et al. 2011). Studies on broilers have shown that dietary composition changes can affect intestinal integrity, morphology, microbiota, and immunity, thereby impacting intestinal health (Fasina et al. 2006; Du et al. 2016). Importantly, the gastrointestinal tract is highly sensitive to stress, which can lead to various damages such as altered intestinal microbiota composition, compromised barrier integrity, and liver injury (Gu et al. 2012; Wu et al. 2022; Sui et al. 2024). In particular, oxidative stress can cause intestinal injury by inducing the production of reactive oxygen species (ROS) and the release of intracellular cytokines (Mitra et al. 2017; Sá et al. 2020). The disruption of intestinal integrity and barrier function not only impairs nutrient digestion and absorption but also allows harmful substances and bacteria to enter the bloodstream, triggering systemic inflammation and worsening intestinal damage (Peterson and Artis 2014; Jarade et al. 2022). Therefore, to enhance growth performance and mitigate morbidity, antibiotics have traditionally been used in broiler production to enhance growth performance and reduce morbidity by preventing and controlling pathogenic microbial infections. However, the emergence of bacterial resistance and public health concerns over antibiotic misuse have led some countries and institutions to impose restrictions or outright bans on antibiotic use in poultry production (Kipper et al. 2022; Chen et al. 2021). Recent findings suggest that improving intestinal antioxidant status by enhancing endogenous antioxidant enzyme activities could be an effective strategy to protect intestinal health and prevent oxidative stress in broilers (He et al. 2017; Yu, Tong, et al. 2022). Natural products, with their diverse biological functions, including antioxidant, anti‐inflammatory, immune‐regulating, and antimicrobial activities, are considered valuable resources for protecting intestinal health (Kim et al. 2020; Todorov et al. 2020; Yu, Wang, et al. 2022).

α‐linolenic acid (ALA, 18:3 n‐3), a ω‐3 polyunsaturated fatty acid (PUFA), is an essential fatty acid mainly present in oil produced from linseed, perilla, rapeseed, and soy (Todorov et al. 2020). ALA is well known for its anti‐inflammatory activity, demonstrated by its ability to interfere with arachidonic acid metabolism and inhibit the prostaglandin biosynthesis pathway, thereby reducing the production of pro‐inflammatory oxylipins (Carroll and Hwang 1980; Caligiuri et al. 2017). In mice with dextran sulfate sodium (DSS)‐induced ulcerative colitis, ALA derived from fermented black radish alleviated inflammation by reducing inflammatory cell infiltration and preserving colon cytoarchitecture (Kim et al. 2020). Todorov et al. (2020) reported that ALA administration altered intestinal microbiota composition and improved intestinal morphology, including elongation of villus structures and an increased abundance of epithelial cells in mice. Similarly, Xie et al. (2022) observed that ALA stimulated villus growth and the proliferation of intestinal stem cells. However, the effects of an ALA‐rich diet on intestinal antioxidant status, morphology, and barrier function of broilers remain unclear. This study hypothesized that ALA could improve intestinal morphology, enhance barrier function, and boost antioxidant status in broilers. Additionally, we further explored the mechanisms by which ALA enhances intestinal barrier function and antioxidant status in broilers.

## 2 Material and Methods

### 2.1 Preparation of α‐Linolenic Acid

The ALA was obtained from Wuhan Cuiyuan Biotechnology Co. Ltd. (CRN0757, Wuhan, China) with an analyzed purity of 99%.

### 2.2 Animals, Experimental Design and Diets

All experimental procedures were approved by the Animal Nutrition Research Institute of Shandong Agricultural University (Taian, Shandong, P. R. China, approval number: SDAUA‐2024‐024). A total of 320 1‐day‐old male Arbor Acres broilers (BW, 39.83 ± 0.82 g) were obtained from Yantai Land Animal Husbandry (Shandong, China) and randomly assigned to four groups based on initial body weight, with 8 replicates of 10 birds per group. The treatment groups received corn‐soybean meal basal diets supplemented with 0 mg (control), 200, 400, and 600 mg of ALA/kg of diet for 42 days, divided into two phases: 1–21 days and 22–42 days. Antibiotic‐free diets were formulated following the Feeding Standard for Chicken of the People's Republic of China (NY/T 33‐2004). The ingredients and nutrient composition of the basal diet are detailed in Table 1. All birds were kept in a temperature‐controlled environment and had free access to feed and water. The temperature was initially set at 35°C on Day 1 and gradually reduced by 0.5°C daily to reach approximately 22°C. The BW and feed intake of birds in units of replicate were recorded weekly to calculate the average daily feed intake (ADFI), average daily gain (ADG), and feed‐to‐gain ratio (F/G).

**TABLE 1:** Ingredients and nutrient composition of the basal diets^a^ (%, as fed‐basis).

Ingredients	Starter (1–21 days)	Grower (22–42 days)
Corn	56.60	55.80
Soybean meal (46% CP)	30.50	25.65
Corn gluten meal	2.50	3.00
Wheat flour	4.00	4.00
Soybean oil	2.00	7.25
Dicalcium phosphate	0.90	0.80
Limestone	1.50	1.50
Premix^b^	2.00	2.00
Nutrient levels		
ME, MJ/kg	12.37	13.11
CP	21.50	19.51
Calcium	0.96	0.84
Total phosphorus	0.66	0.55
Lys	1.45	1.40
Met	0.54	0.50
Thr	0.91	0.80

^a^ The experimental diet was the same basal diet supplemented with 0, 200, 400, and 600 mg of ALA/kg of the basal diet.

^b^ Supplied per kilogram of diet: vitamin A, 11,500 IU; cholecalciferol, 3500 IU; vitamin E, 30 mg; vitamin K_3_, 5 mg; thiamin, 3.38 mg; riboflavin, 9.0 mg; pyridoxine, 8.96 mg; vitamin B_12_, 0.025 mg; choline chloride, 800 mg; calcium pantothenate, 13 mg; niacin, 45 mg; biotin, 0.15 mg; folic acid, 1.20 mg; Mn, 60 mg; Fe, 66.5 mg; Zn, 88 mg; Cu, 8.8 mg; I, 0.70 mg; Se, 0.288 mg.

### 2.3 Sampling

Eight birds (one per replicate) with similar body weights were selected from each group after a 12 h fasting period for sampling. Peripheral blood samples were collected from the wing vein using sterilized needles into tubes coated with coagulant, centrifuged at 3500 *g* for 10 min at 4°C for collection of serum samples, then stored at −80°C for activities of antioxidant enzymes analysis. Subsequently, all of the birds were stunned and sacrificed by cervical dislocation. Approximately 1 cm segments of the mid‐jejunum and ileum were excised and fixed in 4% paraformaldehyde for histomorphological analysis. Additionally, 3 cm segments of the mid‐jejunum and ileum were dissected longitudinally, rinsed with ice‐cold sterile saline to remove contents, frozen in liquid nitrogen, and stored at −80°C for further analysis.

### 2.4 Intestinal Morphology

The paraformaldehyde‐fixed jejunal and ileal segments were dehydrated with graded ethanol, transparentized with xylol, and subsequently embedded with paraffin. Later, the intestinal segments were sliced into 50 μm cross‐sections with a microtome, and finally stained with hematoxylin–eosin (H&E). Ten well‐oriented villi and crypts of each sample were selected for measuring the villus height and crypt depth with a light microscope (Olympus CX31, Tokyo, Japan) and Image‐Pro Plus 6.0 software (Media Cybernetics Inc., Rockville, MD, USA).

### 2.5 Determination of Antioxidant Enzymes in Serum, Jejunum, and Ileum

Frozen jejunal and ileal segments were weighed and homogenized (3 min) with ice‐cold normal saline in the ratio of 1:9 (wt/vol). Subsequently, the homogenates were centrifuged at 3500 *g* for 10 min at 4°C to collect supernatants. The supernatants were diluted to appropriate concentrations to measure antioxidant enzyme activity. The total protein concentration of supernatants was analyzed using bicinchoninic acid (BCA) protein assay kit (Beyotime Institute of Biotechnology, Nanjing, China).

Serum samples and jejunal and ileal supernatants were analyzed for catalase (CAT) activity, total superoxide dismutase (T‐SOD), total antioxidant capacity (T‐AOC), and malondialdehyde (MDA) content using commercial assay kits (Nanjing Jiancheng Bioengineering Institute, Nanjing, China) according to the manufacturer's instructions. Serum antioxidant enzyme activities and MDA concentration were expressed per mL of serum, while results for intestinal tissues were normalized to protein content (per mg) in the jejunum and ileum.

### 2.6 Quantitative Real‐Time PCR Analysis

Total RNA from jejunal and ileal segments was extracted using Trizol reagent (9108, TaKaRa Biotechnology, Dalian, China). The concentration and purity of extracted RNA were measured using a NanoDrop‐100 spectrophotometer (Thermo Fisher Scientific, UK). Reverse transcription PCR was performed to synthesize complementary DNA using the PrimeScriptTM RT reagent kit (RR036A, TaKaRa Biotechnology, Dalian, China). Real‐time PCR was conducted in 96‐well microplates to measure the expression of genes related to jejunal and ileal antioxidant enzymes, the nuclear factor erythroid 2‐related factor 2 (NRF2) signaling pathway, and intestinal barrier integrity using ChamQ SYBR qPCR Master Mix Kit (Q311‐02, Vazyme Biotechnology, Nanjing, China) on an Applied Biosystems 7500 Real‐Time PCR System (Life Technologies, USA). The qRT‐PCR primers for target genes (listed in Table 2) were synthesized by Sangon Biotechnology (Shanghai, China). The 2^−ΔΔ*Ct*^ method was used to calculate the relative mRNA abundance of target genes, normalized to the expression level of the endogenous reference gene *β‐actin*.

**TABLE 2:** Gene‐specific primer sequences for quantitative real‐time PCR.

Gene name^a^	Primer sequence (5′–3′)	GenBank accession number
*β‐actin*	F: ACCGGACTGTTACCAACACC R: CCTGAGTCAAGCGCCAAAAG	NM_205518.1
*IL‐1β*	F: TGCCTGCAGAAGAAGCCTCG R: CTCCGCAGCAGTTTGGTCAT	NM_204524.1
*IL‐4*	F: AGCCTCCACAATTGTTTGGG R: TGAAGTAGTGTTGCCTGCTG	AJ621249.1
*IL‐10*	F: CTGTCACCGCTTCTTCACCT R: GAACTCCCCCATGGCTTTGT	NM_012854.2
*TNF‐α*	F: GAACCCTCCGCAGTACTCAG R: AACTCATCTGAACTGGGCGG	HQ739087.1
*ZO‐1*	F: AGCCCCTTGGTAATGTGTGG R: TTGGGCGTGACGTATAGCTG	XM_015278981.2
*Occludin*	F: ATGCACCCACTGAGTGTTGG R: GAGGTGTGGGCCTTACACAG	NM_205128.1
*CAT*	F: GGTTCGGTGGGGTTGTCTTT R: CACCAGTGGTCAAGGCATCT	NM 001031215.1
*SOD1*	F: TGGCTTCCATGTGCATGAAT R: ACGACCTGCGCTGGTACAC	NM_205064.1
*NRF2*	F: GGGCAAGGCGTGAAGTTTTT R: GGCTTTCTCCCGCTCTTTCT	NM_205117.1
*HO‐1*	F: AGCTTCGCACAAGGAGTGTT R: GGAGAGGTGGTCAGCATGTC	NM_205344.1

Abbreviations: F, forward; R, reverse.

^a^ IL, Interleukin; TNF‐α, Tumor necrosis factor; ZO‐1, Zonula occludens‐1; CAT, Catalase; SOD1, Superoxide dismutase 1; NRF2, Nuclear factor erythroid 2‐related factor 2; HO‐1, Heme oxygenase 1.

### 2.7 Western Blot Assay

Total protein in the jejunum and ileum was obtained with radioimmunoprecipitation assay lysis buffer and protease inhibitor (Beyotime Institute of Biotechnology, Nanjing, China). Subsequently, the nuclear protein was extracted by Nuclear and Cytoplasmic Protein Extraction Kit (Beyotime Institute of Biotechnology, Nanjing, China) for determination of cytoplasmic NRF2 and nuclear NRF2 protein expression. The content of total cellular protein and nuclear protein was detected by the BCA assay kit (Beyotime Institute of Biotechnology, Nanjing, China). Equal amounts of protein were denatured and separated on 10% sodium dodecyl sulfate polyacrylamide gel electrophoresis (SDS‐PAGE), and then electrotransferred onto polyvinylidene difluoride (PVDF) membranes. Later, the membranes were incubated with 5% skimmed milk (w/v) in tris‐buffered saline with 0.1% tween (TBST) buffer for 2 h at room temperature, followed by incubation with primary antibodies against Zonula occludens‐1 (ZO‐1, DF2250; Affinity Biosciences, Jiangsu, China), Occludin (DF7504; Affinity), NRF2 (16396‐1‐AP; Proteintech Group Inc., Wuhan, China), Heme oxygenase 1 (HO‐1, 10,701‐1‐AP; Proteintech), and β‐actin (20536‐1‐AP; Proteintech) overnight at 4°C, and then incubated with secondary antibody for 1.5 h at room temperature. Thereafter, the relative abundance of target proteins was determined with ECL chemiluminescence reagents (E412‐01; Vazyme Biotechnology) and Imager‐Bio‐Rad (Bio‐Rad Laboratories Inc., Hercules, CA, USA), and then quantified by Image J software.

### 2.8 Statistical Analysis

All of the results were analyzed by one‐way ANOVA using the GLM procedure of SAS (SAS institute 2001). The normality and homogeneity of variances were checked before statistical analysis. The data on growth performance parameters was analyzed on a pen basis, whereas other data analysis was based on individual birds. Values were presented as means with a standard error of the mean (SEM). Statistical differences among groups were analyzed by Tukey's HSD test and declared at *p* < 0.05.

## 3 Results

### 3.1 Growth Performance

All birds remained healthy with no recorded mortality. As shown in Table 3, the overall growth performance of birds was not affected (*p* > 0.05) by the inclusion of ALA in either phase or the entire trial period (Days 0–42).

**TABLE 3:** Effects of ALA on the growth performance of broilers^a^.

Items	Dietary ALA concentration, mg/kg				SEM	*p*
	0	200	400	600		
0–21 days						
ADFI, g	37.12	37.43	37.38	37.27	0.168	0.931
ADG, g	29.62	30.33	30.64	30.89	0.225	0.219
F/G, g/g	1.25	1.23	1.22	1.21	0.053	0.474
22–42 days						
ADFI, g	80.12	79.96	80.44	80.01	0.688	0.996
ADG, g	53.82	55.28	55.99	57.03	0.518	0.165
F/G, g/g	1.49	1.45	1.44	1.41	0.018	0.458
0–42 days						
ADFI, g	82.93	82.37	81.25	80.25	0.648	0.495
ADG, g	52.85	54.31	55.03	56.05	0.518	0.168
F/G, g/g	1.57	1.52	1.48	1.44	0.021	0.135
BW, g						
0 day	39.67	40.04	39.67	39.92	0.126	0.363
21 days	621.95	636.89	643.36	648.65	4.733	0.219
42 days	2206.61	2266.64	2295.78	2338.12	21.247	0.165

Abbreviations: ADFI, average daily feed intake; ADG, average daily gain; BW, body weight; F/G, feed/gain.

^a^ Data are means for 8 replicates of 10 birds per replicate.

### 3.2 Intestinal Morphology

**FIGURE 1:**
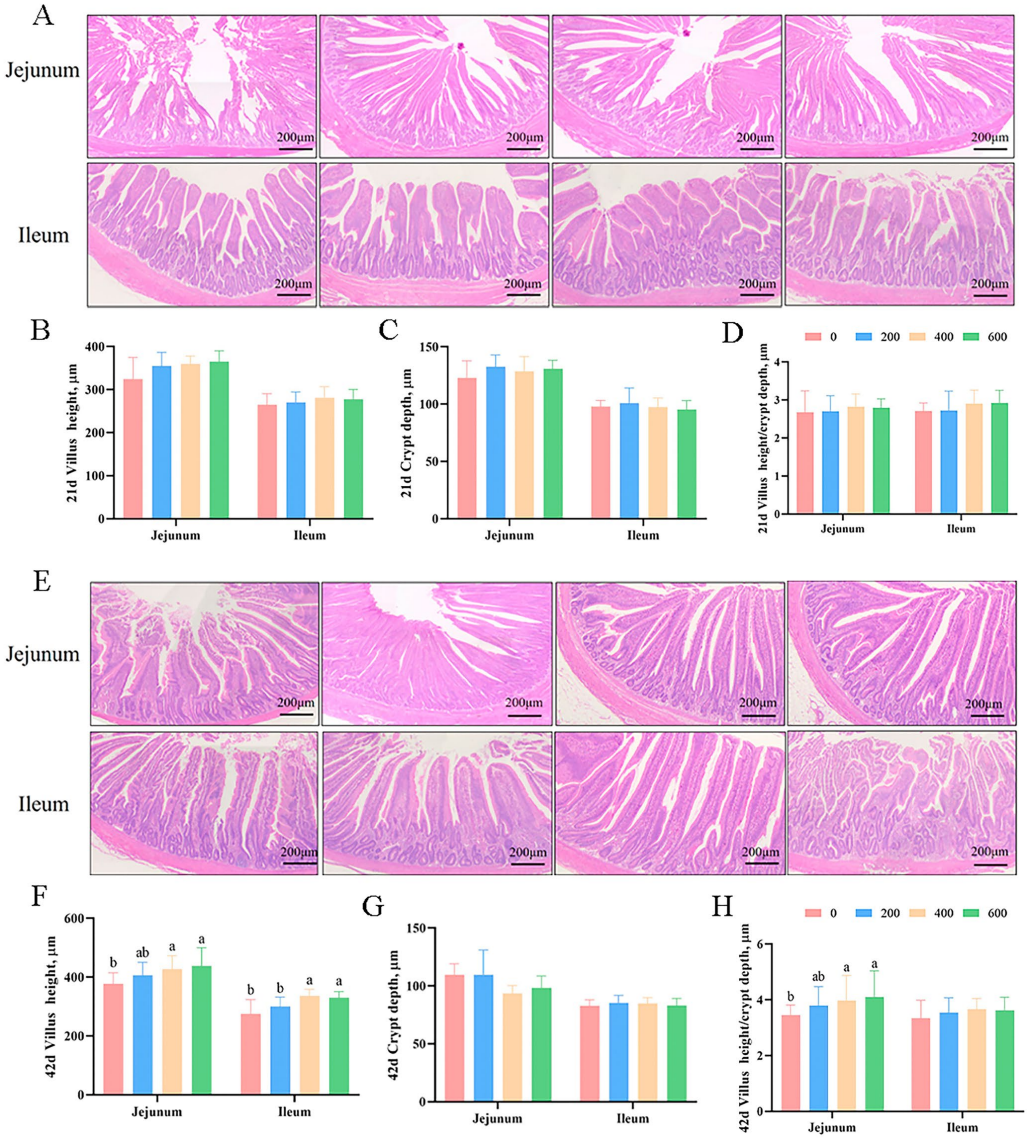
Influence of ALA on the intestinal morphology of broilers. Values are means (*n* = 8), with the standard errors appearing as vertical bars. ^a,b^Means within a row with different letters differ significantly (*P* < 0.05). (A) The images of jejunal and ileal tissues on Day 21; (B–D) the jejunal and ileal villus height, crypt depth, and the ratio of those on Day 21; (E) the images of jejunal and ileal tissues on Day 42; (F–H) the jejunal and ileal villus height, crypt depth, and the ratio of those on Day 42.

Figure 1A–D revealed that dietary ALA supplementation had no significant effects (*p* > 0.05) on villus development, crypt depth, or villus height‐to‐crypt depth ratio in the jejunal and ileal tissues of broilers on Day 21. Figure 1E–H illustrated that ALA supplementation at 400 and 600 mg/kg significantly increased (*p* < 0.05) jejunal and ileal villus height and the jejunal villus height‐to‐crypt depth ratio in broilers on Day 42 compared with the control group. ALA inclusion had no significant effect (*p* > 0.05) on crypt depth in broilers on Day 42.

### 3.3 Expression of Genes and Protein Related to Intestinal Barrier Integrity

**FIGURE 2:**
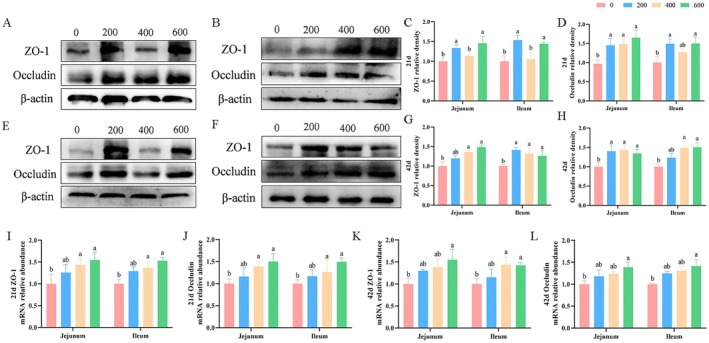
Influence of ALA on the relative protein expression of ZO‐1 and occludin in the jejunum and ileum of broilers. Values were presented as mean with standard errors appearing as vertical bars. ^a,b^Means with different letters differ significantly among the groups (*p* < 0.05). (AH) The jejunal and ileal protein expression of ZO‐1 and occludin (*n* = 3), the jejunal (A, C) and ileal (B, D) protein expression of ZO‐1 and occludin on Day 21, the jejunal (E, G) and ileal (F, H) protein expression of ZO‐1 and occludin on Day 42. (I–L) The jejunal and ileal mRNA expression of ZO‐1 and occludin on Days 21 and 42 (*n* = 8). ZO‐1, Zonula occludens‐1.

As manifested by Figure 2I–L, the results showed that ALA supplementation at 400 and 600 mg/kg significantly increased (*p* < 0.05) jejunal and ileal mRNA expression of ZO‐1 and occludin on Day 21, and ileal ZO‐1 mRNA expression on Day 42 compared with the control group. In addition, compared with the control group, ALA supplemented at 600 mg/kg distinctly increased (*p* < 0.05) the mRNA expression of ZO‐1 in the jejunum and occludin in the jejunum and ileum of broilers on Day 42. Dietary supplementation with 600 mg ALA/kg of diet enhanced (*p* < 0.05) the protein expression of ZO‐1 and occludin in the jejunum and ileum of broilers on Day 21 (Figure 2A–D). Compared with the control group, jejunal and ileal protein expression of ZO‐1 and occludin were both distinctly increased (*p* < 0.05) by ALA administration at 400 and 600 mg/kg of diet of broilers on Day 42 (Figure 2E–H).

### 3.4 Activities of Antioxidant Enzymes

**FIGURE 3:**
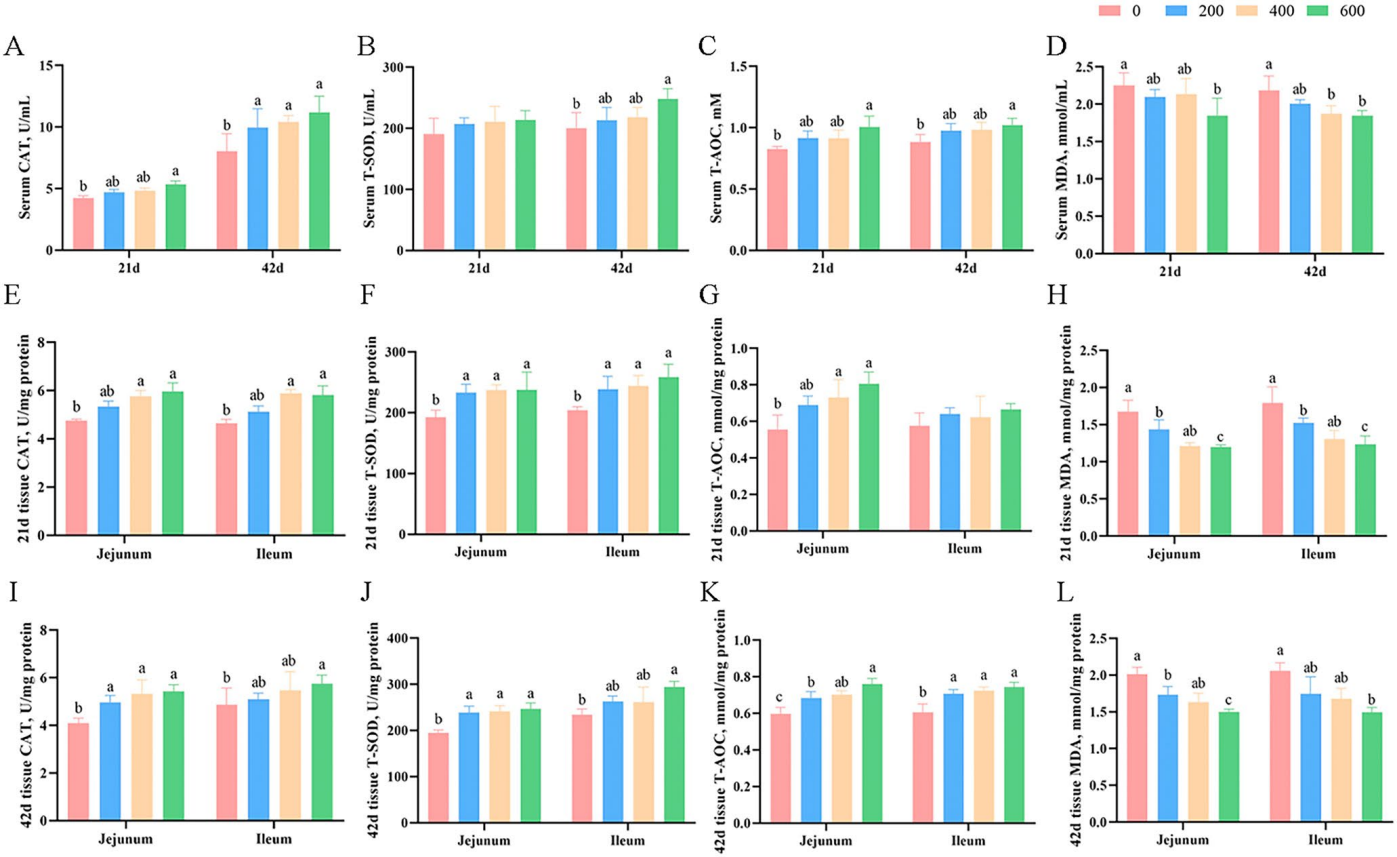
Influence of ALA on the activities of antioxidant enzymes of broilers. Values are means (*n* = 8), with the standard errors appearing as vertical bars. ^a,b^Means within a row with different letters differ significantly (*p* < 0.05). (A–D) Serum activities of antioxidant enzymes of broilers on Day 21; (E–H) The jejunal and ileal activities of antioxidant enzymes of broilers on Day 21; (I–L) The jejunal and ileal activities of antioxidant enzymes of broilers on Day 42. CAT, catalase; MDA, malondialdehyde; T‐AOC, total antioxidant capacity; T‐SOD, total superoxide dismutase.

Broilers fed diets with 600 mg/kg ALA showed significantly higher (*p* < 0.05) serum activities of CAT and T‐AOC on Days 21 and 42, and T‐SOD on Day 42 compared with the control group (Figure 3A–C). ALA supplementation at 600 mg/kg significantly reduced (*p* < 0.05) serum MDA concentration of broilers on Days 21 and 42 (Figure 3D). Without consideration of the supplementation level, broilers fed diets with ALA increased (*p* < 0.05) activities of T‐SOD in the jejunum and ileum on Day 21, T‐AOC in the ileum, CAT, T‐SOD, and T‐AOC in the jejunum on Day 42. Compared with the control group, 600 mg/kg of ALA inclusion increased (*p* < 0.05) activities of CAT and T‐SOD in the jejunum and ileum on Days 21 and 42, T‐AOC in the jejunum on Day 21, and T‐AOC in the jejunum and ileum on Day 42, but decreased (*p* < 0.05) jejunal and ileal MDA concentration on Days 21 and 42 (Figure 3E–L).

### 3.5 Gene Expression of Jejunal and Ileal Antioxidant Enzymes and NRF2 Signaling Pathway

**FIGURE 4:**
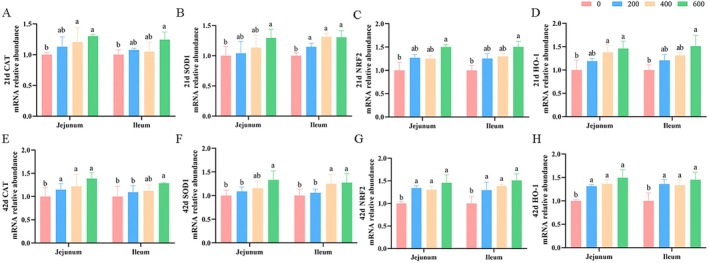
Influence of ALA on the mRNA abundance of antioxidant enzymes and NRF2 pathway in the jejunum and ileum of broilers on Days 21 and 42. Values are means (*n* = 8), with the standard errors appearing as vertical bars. ^a,b^Means within a row with different letters differ significantly (*p* < 0.05). (A–D) The jejunal and ileal mRNA abundance of antioxidant enzymes and NRF2 pathway of broilers on Day 21; (E–H) The jejunal and ileal mRNA abundance of antioxidant enzymes and NRF2 pathway of broilers on Day 42. CAT, catalase; HO‐1, heme oxygenase 1; NRF2, nuclear factor erythroid 2‐related factor 2; SOD1, superoxide dismutase 1.

Dietary ALA supplementation, regardless of level, significantly increased (*p* < 0.05) ileal SOD1 mRNA expression on Day 21, jejunal CAT mRNA expression on Day 42, and NRF2 and HO‐1 mRNA expression in both jejunal and ileal tissues on Day 42 compared with the control group (Figure 4). Moreover, compared with the control group, broilers fed 600 mg/kg of ALA had higher (*p* < 0.05) mRNA expressions of CAT, SOD1, NRF2, and HO‐1 in the jejunum and ileum on Days 21 and 42.

### 3.6 Protein Expression of NRF2 Signaling Pathway

**FIGURE 5:**
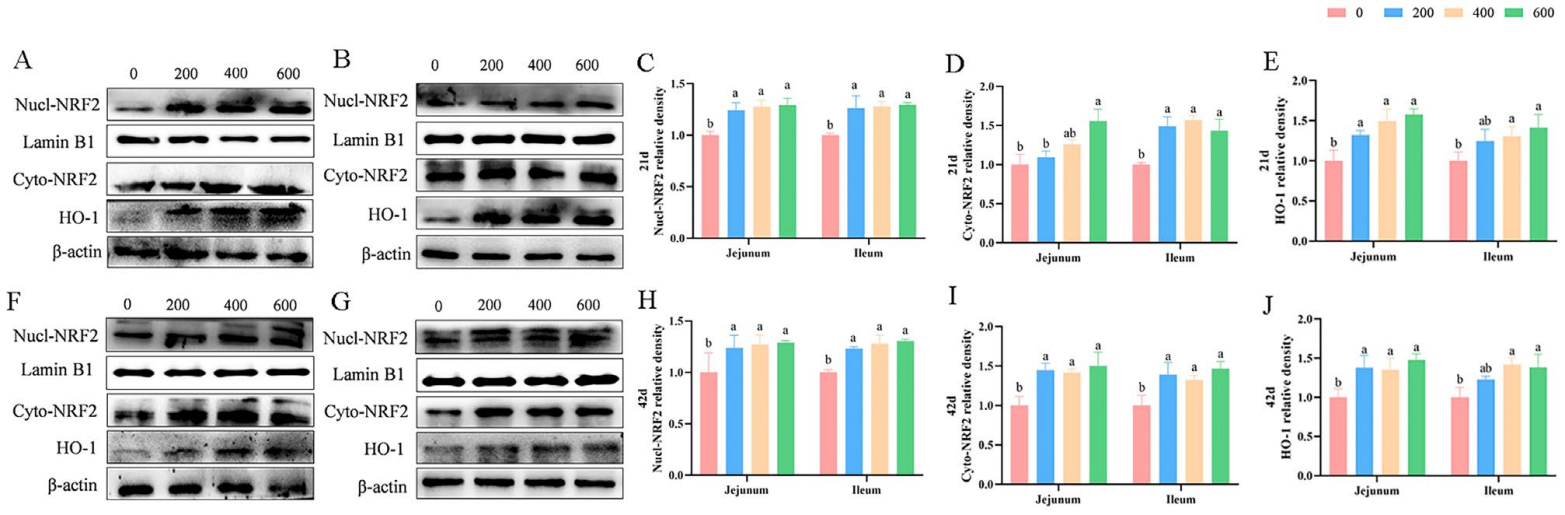
Influence of ALA on the relative protein expression of NRF2 and HO‐1 in the jejunum and ileum of broilers. Values were presented as mean with standard errors appeared by vertical bars (*n* = 3). ^a,b^Means with different letters differ significantly among the groups (*p* < 0.05). (A–E) The jejunal (A) and ileal (B) protein expression of Nucl‐NRF2, Cyto‐NRF2, and HO‐1 on Day 21; (F–J) The jejunal (F) and ileal (G) protein expression of Nucl‐NRF2, Cyto‐NRF2, and HO‐1 on Day 42. Nucl‐NRF2, Nuclear factor erythroid 2‐related factor 2; Cyto‐NRF2, cytoplasmic nuclear factor erythroid 2‐related factor 2; HO‐1, heme oxygenase 1.

As revealed by Figure 5, the results indicated that the protein abundances of jejunal and ileal Nucl‐NRF2 on Days 21 and 42, ileal Cyto‐NRF2 on Day 21, jejunal and ileal Cyto‐NRF2 on Day 42, and jejunal HO‐1 on Days 21 and 42 were up‐regulated (*p* < 0.05) by dietary supplementation of ALA as compared with the control group. Broilers consuming diets with 600 mg/kg of ALA appeared to have the highest protein expressions of Nucl‐NRF2, Cyto‐NRF2, and HO‐1 in the jejunum and ileum on Days 21 and 42.

## 4 Discussion

A healthy intestinal environment is essential for nutrient absorption and innate defense against pathogen infections (Chairakaki and Greece 2009; Xie et al. 2011). Experimental evidence has shown the anti‐inflammatory and antioxidant properties of ALA in both in vitro and in vivo studies (Hassan et al. 2010; Reifen et al. 2015; Pauls et al. 2018; Kim et al. 2020). Furthermore, growing evidence suggests that ALA holds potential in alleviating intestinal inflammatory disease phenotypes (Pearl et al. 2014; Reifen et al. 2015; Wang et al. 2022). However, the effects of ALA on gut homeostasis and intestinal health in broilers under normal feeding conditions remain largely unknown. Taken together, this study investigated the protective effects of ALA on intestinal health by examining intestinal morphology, barrier function, and antioxidant status in broilers.

The results of this study showed that ALA administration did not significantly affect ADFI, ADG, and F/G in broilers. No evidence was found to indicate positive effects of ALA on broiler growth performance. Notably, the animal age, diet type, experimental conditions, hygiene, and altered gut microbiota may also affect the performance response of broilers to dietary components (Goel et al. 2008). Therefore, further research is needed to characterize the effects of ALA on broiler growth performance.

Interestingly, we found that ALA supplemented at 400 and 600 mg/kg increased the jejunal and ileal villus height, as well as the ratio of jejunal villus height to crypt depth in broilers on Day 42, compared with the control group. Consistent with our findings, a previous study investigated the effects of ALA inclusion on intestinal morphology and gut microbiota composition in mice. It found that ALA‐containing diets increased villus length and mucosal thickness (Todorov et al. 2020), supporting the notion that ALA may enhance intestinal mucus secretion and nutrient absorption surface area. Additionally, this study showed that dietary ALA supplementation at 400 and 600 mg/kg increased jejunal and ileal mRNA expression of ZO‐1 and occludin on Day 21, as well as ileal mRNA and protein expression of ZO‐1 and occludin on Day 42, compared with the control group. The intestinal mucosal barrier, which relies on a layer of epithelial cells and tight junction proteins (including ZO‐1 and occludin), serves as the primary innate defense against pathogen invasion (Chairakaki and Greece 2009; Xie et al. 2011; Broom and Kogut 2018). Xie et al. (2022) demonstrated that ALA, mediated by *Ruminococcaceae*, promotes the proliferation of intestinal stem cells and villus growth in mice. This suggests that ALA has positive effects on intestinal renewal, regeneration, and potentially preventing intestinal inflammation. In summary, this study indicates that dietary ALA supplementation may enhance intestinal health by strengthening the mucosal barrier and improving villus morphology.

Numerous studies have highlighted the protective effects of ALA in managing inflammatory bowel disease (Hassan et al. 2010; Reifen et al. 2015). Specifically, Tyagi et al. (2012) reported that replacing part of dietary linoleic acid with ALA reduced colonic inflammation by suppressing pro‐inflammatory mediators in a rat model of experimental colitis. Kim et al. (2020) also observed that ALA supplementation alleviated DSS‐induced ulcerative colitis by reducing colon damage, crypt shortening, and pro‐inflammatory macrophage populations in the colon. Similar to that, the anti‐inflammatory effects of ALA were exhibited in pro‐inflammatory macrophages associated with enhanced oxylipin secretion (Pauls et al. 2018). Importantly, oxidative stress can lead to intracellular cytokine production, contributing to chronic and systemic inflammation (Dongare et al. 2012; Mitra et al. 2017). Erdinest et al. (2012) demonstrated that ALA exerts anti‐inflammatory effects by inhibiting ROS generation. Notably, ROS is considered a key trigger for oxidative stress and intestinal injury (Su et al. 2018; Sá et al. 2020). Growing evidence suggests that enhancement of endogenous antioxidant enzyme activities could reduce the excessive production of ROS and further relieve oxidative stress (He et al. 2017; Su et al. 2018). Thus, the antioxidant status of the body can be assessed by measuring the activity of antioxidant enzymes such as CAT, T‐SOD, and GSH‐Px. In this study, supplementation with 600 mg/kg ALA enhanced the activity of serum CAT and T‐AOC, intestinal CAT and T‐SOD, and upregulated intestinal mRNA expression of CAT and SOD1, while reducing MDA concentration in broilers. This suggests that ALA can improve intestinal antioxidant status by enhancing the activity of intestinal antioxidant enzymes. Furthermore, we demonstrated that supplementation with 600 mg/kg ALA up‐regulated jejunal and ileal mRNA and protein expression of NRF2 and HO‐1 in broilers. NRF2 is a key transcription factor that maintains cellular redox balance and regulates the body's antioxidant status (Johnson et al. 2008). Upon activation, NRF2 dissociates from Keap1, translocates to the nucleus, and binds to antioxidant response elements to initiate the transcription of antioxidant genes. These findings suggest that ALA enhances intestinal antioxidant status by increasing the activity of intestinal antioxidant enzymes and activating the NRF2 signaling pathway. Supporting our findings, Yu et al. (2013) demonstrated that ALA exerted cardioprotective effects against doxorubicin‐induced cardiotoxicity in rats by enhancing the antioxidant defense system via NRF2 activation and increasing ventricular SOD and CAT activities. While limited information exists on the effect of ALA on intestinal antioxidant status, this study presents a promising strategy for enhancing intestinal health by strengthening the antioxidant status in broilers. The study conducted by Pauls et al. (2018) indicated that the anti‐inflammatory activity of ALA was likely attributed to the production of oxylipins. Oxylipins are derived from omega‐3 PUFAs, including ALA. The mechanisms by which ALA has anti‐inflammatory potential are thought to be likely mediated by oxylipin production. Therefore, further research is needed to elucidate the mechanisms by which ALA exerts its intestinal antioxidant effects in broilers.

The present study revealed that dietary inclusion of 600 mg/kg ALA significantly improved intestinal barrier function, morphology, and antioxidant status in broilers. Kim et al. (2014) summarized that ALA is generally considered safe, with no restrictions on its use. In contrast, Regensburger et al. (2012) reported that ALA, like other fatty acids, can produce lipid peroxidation products when exposed to air, potentially causing adverse effects at improper dosages. Information on the effects of ALA inclusion on animal intestinal health remains limited. Further research is needed to clarify the optimal concentration of ALA for improving broiler intestinal health.

## 5 Conclusions

Dietary supplementation with 600 mg/kg ALA enhanced intestinal barrier function, intestinal morphology, and antioxidant status in broilers, indicating a promising potential of ALA‐based protection for broiler intestinal health.

## Author Contributions

**Ao Kang:** data curation (equal), methodology (equal), writing – original draft (equal). **Jialei Ni:** conceptualization (equal), data curation (equal), methodology (equal), resources (equal). **Xinyu Cheng:** conceptualization (equal), investigation (equal), methodology (equal). **Shuyu Wu:** resources (equal), software (equal), validation (equal). **Yun Liu:** funding acquisition (equal), validation (equal), writing – review and editing (equal). **Weiming Ma:** project administration (equal), writing – review and editing (equal). **Dong Wang:** funding acquisition (equal), project administration (equal), writing – review and editing (equal).

## Conflicts of Interest

The authors declare no conflicts of interest.

## Data Availability

The data that support the findings of this study are available on request from the corresponding author.
